# Assessing Heterogeneity in Sentiment Changes in Text-Based Counseling: Latent Class Trajectory Analysis

**DOI:** 10.2196/75091

**Published:** 2025-09-05

**Authors:** Ziru Fu, Yu Cheng Hsu, Christian Shaunlyn Chan, Paul Siu Fai Yip

**Affiliations:** 1 Department of Social Work and Social Administration, Faculty of Social Sciences, Faculty of Social Sciences, University of Hong Kong, Hong Kong, China (Hong Kong); 2 The Hong Kong Jockey Club Centre for Suicide Research and Prevention, University of Hong Kong, 5 Sassoon Rd, Sandy Bay, Hong Kong, 999077, China (Hong Kong), 852 2831 5232; 3 Department of Psychology and Linguistics, International Christian University, Tokyo, Japan

**Keywords:** text-based counseling, latent class trajectory analysis, sentiment analysis, growth mixture model, multinomial logistic regression

## Abstract

**Background:**

Online text-based counseling services are becoming increasingly popular. However, their text-based nature and anonymity pose challenges in tracking and understanding shifts in help-seekers’ emotional experience within a session. These characteristics make it difficult for service providers to tailor interventions to individual needs, potentially diminishing service effectiveness and user satisfaction.

**Objective:**

This study aimed to identify distinct within-session sentiment trajectories among help-seekers in online text-based counseling and examine key variables associated with trajectory membership.

**Methods:**

A total of 6207 counseling sessions were randomly extracted from an online text-based counseling service in Hong Kong. A latent class trajectory analysis of help-seekers’ in-session sentiment was conducted using a growth mixture model (GMM) to identify latent groups of help-seekers exhibiting specific sentiment trajectories. Sentiment scores of help-seeker messages, labeled by ChatGPT, served as the primary variable for trajectory modeling. Subsequently, a multinomial logistic regression was performed to identify variables associated with class membership.

**Results:**

The GMM identified 3 distinct sentiment trajectories as the best fit: (1) steady improvement (1171/6207, 18.9%), (2) deterioration (1119/6207, 18.0%), and (3) dip-then-rebound (3917/6207, 63.1%). Compared with the Dip-Then-Rebound Class, help-seekers in the Deterioration Class were more likely to report suicidal ideation (OR=1.28, 95% CIs 1.07-1.52, *P*=.006), present with family (OR=1.56, 95% CIs 1.19-2.08, *P*=.002) or physical health-related concerns (OR=1.67, 95% CIs 1.02-2.74, *P*=.04), have an unknown gender status (OR=1.32, 95% CIs 1.04-1.67, *P*=.02), access the service through the anonymous channel (OR=1.30, 95% CIs 1.03-1.63, *P*=.03), depart from the session prematurely (OR=9.76, 95% CIs 8.33-11.36, *P*<.001), and have shorter session durations (OR=0.77, 95% CIs 0.71-0.84, *P*<.001).

**Conclusions:**

We identified 3 distinct trajectories of help-seekers’ in-session sentiment. Identifying the most likely trajectory at an early stage in the session could potentially help counselors adjust their approaches, thereby improving the effectiveness of text-based counseling and enhancing help-seeker satisfaction.

## Introduction

The demand for mental health services has surged in recent years [1], driving the rapid expansion of online text-based counseling [2]. This modality reduces barriers commonly associated with traditional face-to-face services [3] by allowing help-seekers to communicate with counselors in real-time at any location, often anonymously, which may lower fear of being stigmatized and increase openness in disclosing concerns [4].

Despite its benefits, the anonymity inherent in text-based counseling poses challenges in gathering useful help-seeker information and accurately assessing therapeutic progress [5,6]. In addition, the lack of nonverbal cues in text communication can lead to misunderstandings and hinder counselors from readily evaluating the severity of issues [7]. These difficulties highlight the need for alternative approaches to better understand help-seekers’ emotional processes and improve the effectiveness of counseling services [8].

Natural language processing (NLP) integrates linguistics and artificial intelligence (AI) to analyze human language recorded in text or audio [9]. Within NLP, sentiment analysis focuses on classifying expressed emotions (eg, positive, neutral, or negative) [10]. In the context of counseling research, sentiment analysis can serve as a supplementary tool for tracking help-seekers’ emotional status and therapeutic process [10], offer an objective indicator of session outcomes that circumvents the recall bias inherent in self-report methods, and efficiently process large volumes of text. Previous research [eg, 10-12] has recommended using sentiment analysis to track help-seekers’ moment-to-moment shifts in emotional experiences and investigate how these shifts relate to counseling outcomes.

Recent studies have applied sentiment analysis to gain insight into the counseling process. For instance, Syzdek [13] used the syuzhet package in R (R Foundation for Statistical Computing) [14] to label the sentiment of both client and therapist messages as positive, neutral, or negative across 20 psychotherapy sessions. A hierarchical linear model revealed that client sentiment trended more negatively within each session but became more positive over multiple sessions, and that clients and therapists seemed to mirror each other’s sentiment. However, a lack of formal outcome measures prevented the exploration of the sentiment-outcome relationship.

Althoff and colleagues [15] analyzed crisis helpline interactions using the Linguistic Inquiry and Word Count (LIWC) tool to label help-seeker messages as positive, neutral, or negative. Help-seekers’ postsession self-reported feelings (“Better,” “Same,” or “Worse”) served as a proxy for session quality. Sessions with response “Better” were coded as high-quality sessions, and sessions with feedback “Same” or “Worse” were coded as low-quality sessions. The authors found that help-seekers tended to start sessions with high-level negativity, but the sentiment became more positive over time for both high- and low-quality sessions.

Despite these achievements, previous studies relied on lexicon-based approaches (eg, syuzhet package in R and Linguistic Inquiry and Word Count [LIWC]), which tend to overlook context-dependent nuances in language [12]. In addition, while research has demonstrated that help-seekers’ sentiment can fluctuate throughout a session, less attention has been given to the underlying factors that drive or contribute to these emotional shifts. Help-seekers are not a homogeneous group; their emotional trajectories are likely shaped by individual characteristics and contextual factors. Identifying variables associated with sentiment trajectory membership is crucial because certain help-seekers—such as those reporting suicidal ideation or accessing services anonymously—may be at higher risks of deterioration and may require different intervention strategies [5].

A few studies found that in-session sentiment changes are associated with various clinically meaningful indicators and demographic differences. Stamatis et al [16] demonstrated that text sentiment patterns are prospectively associated with symptoms of depression, generalized anxiety, and social anxiety, with distinct linguistic markers characterizing each condition. This underscores the importance of accounting for presenting issues when modeling emotional shifts. Similarly, Eberhardt et al [10] found that sentiment trends in psychotherapy sessions were significantly correlated with self-reported distress levels, highlighting the relationship between distress levels and unique sentiment trajectories. Demographic characteristics have been linked to differences in emotional expression and engagement in the counseling process. Efe et al [17] found that linguistic markers exhibited gender differences, with female users more frequently using negative emotion words, which were predictive of psychiatric symptoms. Further investigation into the clinical and demographic factors associated with sentiment changes could extend the use of sentiment analysis, ultimately enhancing counseling practices.

In this study, we aimed to identify distinct sentiment trajectories among help-seekers and examine whether psychological profiles (eg, presenting problems and baseline level of distress), demographic characteristics (eg, gender), and help-seeker behaviors (eg, session duration and means of access) correlated with the evolution of their sentiments within a session. By uncovering latent sentiment trajectory classes and identifying correlates of trajectory membership, we sought to develop more tailored interventions and service planning strategies to improve counseling effectiveness and help-seeker satisfaction.

To achieve this, we applied latent class trajectory analysis (LCTA) to identify distinct sentiment trajectories in Open Up, an online text-based counseling service in Hong Kong. Fu et al [11] reported that GPT-3.5 and GPT-4 significantly outperformed both lexicon-based and other machine learning methods in classifying text sentiment from Open Up. Building on this finding, we used GPT-4 to label each help-seeker message. Subsequently, multinomial logistic regression was used to reveal whether and to what extent different variables—including demographic information, presenting problems, and distress levels—were associated with membership in each trajectory class. By integrating advanced NLP techniques with trajectory analysis, this study sought to enhance our understanding of help-seekers’ in-session emotional shifts and their implications for effective online counseling.

## Methods

### Data Source

This study analyzed counseling transcripts from Open Up, a 24/7 online text-based counseling platform in Hong Kong providing support to youth with various issues, including mental health problems, family relationships, and academic pressure [18]. Open Up was launched in October 2018 as the first known online text-based counseling platform in Hong Kong [19]. The service is free of charge and accessible through multiple channels, including the official web portal, SMS text-messaging, WhatsApp (Meta), Facebook (Meta), and WeChat (Tencent). Help-seekers can communicate anonymously with a counselor or trained volunteer in real time.

The platform is staffed by licensed professional social workers and trained volunteers. On average, counselors have eight years of professional experience, including 1 to 2 years in online counseling. Most volunteers are undergraduate students studying social work, psychology, or counseling and must complete a rigorous training program consisting of a minimum of 45 hours of structured instruction and 36 hours of evaluated practice sessions under the supervision of experienced staff counselors.

From October 2018 to July 2024, Open Up delivered 169,787 valid counseling sessions to over 60,000 unique help-seekers, making it the largest synchronous text-based counseling and crisis intervention service in Hong Kong. Valid sessions are defined as those comprising at least 4 message exchanges between the help-seeker and the counselor, in line with criteria established in a previous study [18]. This threshold was adopted because some users logged on to the platform without revealing any intention to use the service; such sessions are typically briefer than 4 message exchanges.

The platform has earned widespread recognition and popularity among youth, educators, and mental health professionals. According to a postsession survey (20.7% of users, n=35,235; [20]), 83.0% reported that they found the session helpful. These figures attest to Open Up’s reputation as a trusted, accessible, and effective resource for professional youth mental health support in Hong Kong.

### Procedure

**Figure 1.**
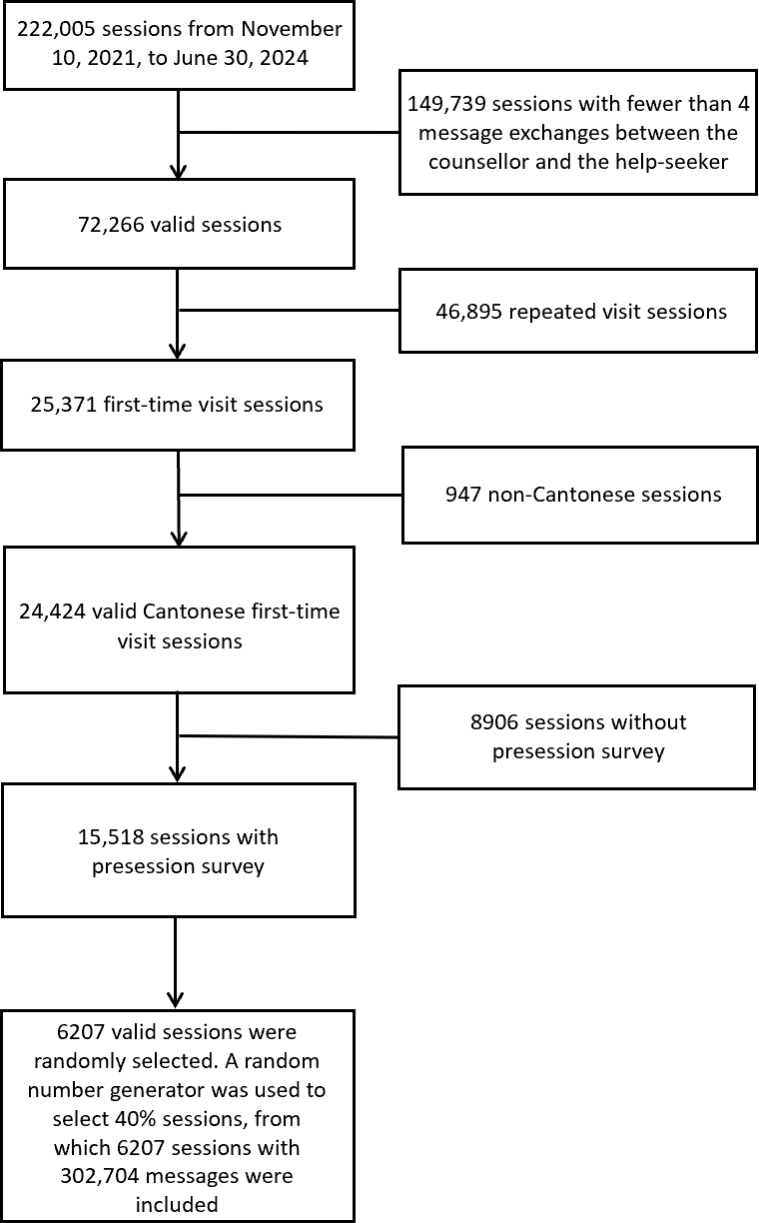
Data inclusion.

As illustrated in Figure 1, we initially extracted 222,005 counseling sessions from November 10, 2021 (when the presession survey was introduced) to June 30, 2024 (our chosen cutoff for data analysis).

We excluded 149,739/222,005 sessions (67.4%) with fewer than 4 message exchanges between the counselor and help-seeker, as these were too brief for sentiment analysis. This exclusion left 72,266/222,005 valid sessions (32.6%).

Next, we excluded non-first–time visit sessions, retaining 25,371 sessions (35.1% of 72,266) in which help-seekers used Open Up for the first time. Focusing on first-time visits prevents potential confounds introduced by repeated sessions, in which counselors may already know help-seekers’ profiles and presenting problems. Over multiple visits, help-seekers may also become more comfortable or adopt different communication patterns, and counselors might tailor their approach based on previous interactions. By restricting the analysis to first-time visits, we ensure that (i) all sessions represent an initial point of contact without previous counselor knowledge, (ii) help-seekers’ emotional trajectories are observed from a more uniform “starting point,” and (iii) to the best of our knowledge, the independence of observations is maintained. This approach increases the clarity and comparability of the data when examining sentiment trajectories and associated outcomes. We then removed 947 sessions (3.7% of 25,371) that were non-Cantonese, resulting in 24,424 valid Cantonese first-time sessions.

Among these sessions, we included only those with presession surveys that provided information about help-seekers’ primary concerns, level of psychological distress, and suicide ideation. This retained 15,518 (63.5% of 24,424) sessions. The remaining 8906/24,424 sessions (36.5%) were excluded due to missing presession survey data. As the presession survey was randomly distributed to help-seekers in Open Up and completion of the survey was voluntary, this exclusion could introduce selection bias if survey participation correlated with specific session or help-seeker characteristics. To assess this possibility, we compared the available metadata (communication channel, premature departure, repeated help-seeker, queue time, session duration, age group, and gender) between included and excluded sessions, as presented in Table S1 in Multimedia Appendix 1.

Taking into consideration computational and resource constraints, we randomly selected 40% (6207/15,518) of these sessions using Python package pandas (version 1.5.3; Python Software Foundation). Specifically, the sample method was applied with the fraction parameter set as 0.4 and the fixed random seed assigned as 42 to ensure reproducibility. These 6207 sessions constituted the final dataset for trajectory modeling.

### Measurements

#### Presession Survey for Help-Seekers’ Psychological Distress Profile

The presession survey contained 8 questions that assessed help-seekers’ psychological distress. It included three components: (i) help-seeker’s primary concern, (ii) severity of psychological distress, and (iii) self-harm and suicide ideation.

Primary concern was assessed with the question, “What is the issue that concerns you the most?” Respondents were given 14 options to choose from: (1) mental health, (2) family relation, (3) intimate relationship, (4) interpersonal relationship, (5) study, (6) career, (7) traumatic experience, (8) physical health, (9) personal development, (10) sexual orientation/gender-related distress, (11) addictive behavior, (12) social unrest, (13) COVID-19 pandemic, and (14) others. Help-seekers can only choose 1 option. For analysis, the primary issue was treated as a categorical variable (coded 1 to 14).

Psychological distress was measured using the 6-item Kessler Psychological Distress Scale (K6). The K6 is a widely used tool for screening psychological distress [21]. It measures the frequency of 6 distress-related symptoms over the past month, including feelings of nervousness, hopelessness, restlessness, depression, worthlessness, and fatigue [22,23]. The K6 has been shown to have strong reliability and validity to screen for psychological distress among young people in Hong Kong [24,25]. Each of the 6 items was scored from 0 to 4, yielding a total possible score of 0 to 24 (with higher scores indicating greater distress). Self-harm and suicidal ideation were assessed with one question, “In the past two weeks, have you had thoughts about hurting or killing yourself?” The response was treated as a binary variable (yes=1, no=0).

#### Help-Seeker Behaviors

Variables related to help-seekers’ behaviors were collected using both automatic system recording and manual coding, depending on the variable.

##### Communication Channel

The mode by which help-seekers accessed the service was recorded as “Anonymous channel” (coded as “1”) that is, the web portal, where no personal identifier was available, or “Non-anonymous channel” (coded as “0”), including WhatsApp, Facebook Messenger, or WeChat, where help-seekers might have identifiable information such as their account numbers. The communication channel was automatically recorded by the online system as part of the session metadata.

##### Repeated Help-Seeker

This variable indicated whether the help-seeker used Open Up again after their first visit, with one-time help-seekers coded as “0” and repeated help-seekers coded as “1.” Repeated help-seeker status was determined by linking sessions from each unique help-seeker using system-generated identifiers: for web portal users, this was based on a hashed version of IP address; for nonweb portals (eg, Facebook, WhatsApp), on a hashed version of social media account numbers.

##### Premature Departure

It was measured whether the help-seeker ended the session abruptly without indicating their intention to leave or establishing a mutual agreement with the counselor to terminate the session. A session was coded as “1” if the help-seeker departed prematurely; otherwise, it was coded as “0.” Premature departure was coded according to the logic-based rules outlined in Xu et al [26].

##### Queue Time

It indicates the time (in minutes) a help-seeker waited in the queue before a counselor picked up the session. Queue time was calculated from system timestamps of when the help-seeker joined the queue and was connected to a counselor, both of which were automatically recorded.

##### Session Duration

It refers to the time (in minutes) from when the help-seeker was connected to a counselor until the end of the session. Session duration was calculated from system timestamps of when the help-seeker was connected to a counselor and when the session ended, both of which were automatically recorded.

### Counselors’ Postsession Summary for Help-Seekers’ Demographic Information

After each session, the counselor recorded basic demographic information about the help-seeker, including age group (eg, secondary school student, university student, nonstudent youth, middle-aged, and unknown), and gender (male, female, and unknown). Age group was coded as “1” for secondary school students, “2” for university students, “3” for non-student youth, “4” for middle-aged individuals, and “5” for unknown. Similarly, gender was coded as “1” for male, “2” for female, and “3” for unknown. There was no missing data in any of the variables used for trajectory modeling or regression analysis.

### Sentiment Label

Sentiment scores of help-seeker messages, labeled by GPT-4 (via ChatGPT), served as the primary input for sentiment trajectory modeling. Following the procedure in Fu et al [11], each help-seeker message was classified as positive, neutral, or negative. GPT-4 demonstrated high performance in classifying Cantonese text-based counseling messages, with an accuracy of 95.3% (5880/6169) and an *F*_1_-score of 94.0%, outperforming traditional machine learning approaches.

According to the codebook established in Fu et al [11], positive messages typically expressed hope, gratitude, or encouragement (eg, “You’ve done very well”; “I really want to change”); negative messages conveyed complaint, distress, or hopelessness (eg, “I’m depressed and frustrated all day”; “Nobody likes me”); and neutral messages consisted of factual or procedural content, such as greetings or demographic disclosures (eg, “I’m 25 y old”; “I’ve been dating my boyfriend for several months”). The labeling was conducted using the OpenAI GPT-4 API via Python package openai. The model’s parameter setting is outlined in Table 1, which followed those of Fu et al [11].

The labeling was conducted using the OpenAI GPT-4 API via Python package *openai* (version 1.23.2; Python Software Foundation). The model’s parameter setting is outlined in Table 1, which followed those of Fu et al [11].

A systematic review of sentiment analysis indicates that positive texts are often coded as 1, neutral texts as 0 and negative texts as −1 [27]. This study followed the same scoring approach.

**Table 1.** GPT-4 prompt settings.

Model	GPT-4
prompt	“Is the sentiment of this Cantonese text positive, neutral, or negative? Respond with a sentiment label only.”
max_token	8192
engine	gpt-4
temperature	0.0

### Statistical Analysis

#### Average Sentiment Score for Each Epoch in Each Session

To analyze sentiment changes over the course of each counseling session, we split sessions into 5 equal segments (hereafter referred to as “epochs”) based on the total number of message exchanges. The decision was informed by previous studies that analyzed counseling stages and session progression [eg, 15,28,29]. For instance, Pérez-Rosas et al [28] explored the change of linguistic features (eg, linguistic alignment between the counselor and the help-seeker, sentiment features, topics discussed during the session) as a counseling session progressed. They split each session into 5 stages of nearly identical number of message exchanges between the counselor and the help-seeker [28]. We opted for a similar approach that allowed us to approximate the session progression without assigning messages into specific stages and ensured all sessions were structured consistently, which could increase the efficiency of trajectory modeling and improve the interpretability of trajectories.

After dividing each session into 5 equal epochs, we computed an average sentiment score for each epoch. Mathematically, for the j-th epoch of the i-th session, the average sentiment score $S_{i,j}$ was given by:


$$S_{i,j}=\frac{1}{N_{i,j}}\sum_{k=1}^{N_{i,j}}s_{i,j,k}$$

where $N_{i,j}$ is the number of messages in the j-th epoch of the i-th session. $s_{i,j,k}$ is the sentiment score of the k-th message in the j-th epoch of the i-th session.

#### Latent Class Trajectory Analysis

The Growth Mixture Model (GMM) was used to model the help-seekers’ sentiment trajectory throughout the session. We chose GMM due to its ability to capture heterogeneity in patterns of change and allow for random effects of individual differences [30]. Each latent class comprises sessions with similar patterns of sentiment change. To account for the nonlinear nature of counseling dynamics including sentiment change [31], we included quadratic terms in our model. For each latent class C, the estimated score S is:


$$g\left(S\right)=b_{0}^{c}+b_{1}^{c}X+b_{2}^{c}X^{2}$$

where $b_{0}^{c}$ is the random intercept for class C, so it is the sum of the mean intercept of that class and the random error $\epsilon_{i}$ for each session i. X and $X^{2}$ represent the linear and quadratic terms of the epoch, respectively. $b_{1}^{c}$ and $b_{2}^{c}$ are the estimated slopes for the linear and quadratic terms, respectively. Because sentiment scores naturally range from −1 to 1, a beta link function was applied to ensure predictions remained within these bounds. The GMM estimated the likelihood of each session belonging to each latent class; each session was then assigned to the class with the highest likelihood.

Because sentiment scores naturally range from −1 to 1, a beta link function was applied to ensure predictions remained within these bounds.

GMM was implemented using R package lcmm (version 2.1.0; R Core Team; [32]) and estimated iteratively with the number of latent classes ranging from 1 to 6. The lower bound (1-class model) served as the baseline, representing a homogeneous population without trajectory heterogeneity. The upper bound (6-class model) was chosen based on both previous literature and practical considerations: First, previous studies employing latent class trajectory or GMM approaches in counseling or psychotherapy contexts typically identified up to 4 or 5 classes [33-35]. The 6-class model was included as a conservative upper limit to ensure all plausible models were evaluated. Second, this range allowed sufficient flexibility to detect meaningful heterogeneity while minimizing the risk of overfitting or generating very small classes (ie, <5% of the sample), which are often unstable and difficult to interpret. The optimal number of classes was determined based on a combination of fit indices, including the lowest Akaike’s Information Criterion (AIC), lowest Bayesian Information Criterion (BIC), and the highest maximum log-likelihood.

#### Associations of Variables With Trajectory Class

Multinomial logistic regression was applied to identify variables associated with trajectory class membership. The membership of latent groups of sentiment trajectory derived from the GMM was treated as the outcome variable. Predictors included help-seekers’ psychological distress profile (ie, K6 distress scores, primary issue, and suicidal ideation), help-seekers’ behaviors (eg, communication channel and premature departure), and help-seekers’ demographic information (ie, age group and gender). Regression was conducted using Python package statsmodels (version 0.13.5; Python Software Foundation; [36]). The resulting coefficients and *P* values of the predictors were evaluated for statistical significance and interpretation of their relationships with different sentiment trajectory classes.

### Ethical Considerations

The study was conducted by researchers at The University of Hong Kong, who also served on the Evaluation and Knowledge Dissemination Subcommittee in Open Up, the organization that provided the data. Ethical approval was obtained from the university’s Human Research Ethics Committee (reference number: EA230548). Before using the Open Up service, users consented to the use of their data for research purposes by accepting the platform’s Terms of Service. Counselors also provided written informed consent. No compensation, financial or otherwise, was provided to participants. Each user was identified through a hashed version of their IP address or social media account. All personally identifiable information disclosed during sessions was anonymized and deidentified to protect user privacy and ensure confidentiality.

## Results

### Data Descriptives

The randomly selected 6207 sessions contained a total of 302,704 messages, including 151,668 messages from help-seekers. As exhibited in Table 2, GPT-4 labeled 13,777/151,668 (9.1%) of these help-seeker messages as positive, 100,536/151,668 (66.3%) as negative, and 37,355/151,668 (24.6%) as neutral.

Table 3 presents the help-seekers characteristics for the 6207 sessions. A total of 1965/6207 help-seekers (31.7%) reported mental health as their primary concern. Help-seekers reported a mean K6 score of 14.53 (SD 4.61), indicating moderate distress. In the past 2 weeks, 2123/6207 help-seekers (34.2%) had thoughts of self-harm or suicide. Regarding demographics, 2097/6207 help-seekers (33.8%) were identified as nonstudent youth, and 3460/6207 (55.7%) were female. Most help-seekers (5212/6207, 84.0%) accessed Open Up via the web portal. Furthermore, 2380/6207 help-seekers (38.3%) returned to Open Up after their initial visit, while 1773/6207 (28.6%) left the session prematurely. On average, help-seekers waited 13.04 minutes (SD 14.71) for a counselor and sessions lasted 63.31 minutes (SD 30.89).

**Table 2.** Number and percentage of messages with each sentiment label.

Sentiment label	Sentiment score	Messages in total, n (%)	Help-seeker messages, n (%)
Positive	1	29,452 (9.7)	13,777 (9.1)
Negative	−1	135,292 (44.7)	100,536 (66.3)
Neutral	0	137,960 (45.6)	37,355 (24.6)

**Table 3.** Descriptives of help-seeker characteristics.

Characteristics	Values
Primary concern, n (%)	
Mental health	1965 (31.7)
Family relationship	564 (9.1)
Intimate relationship	953 (15.4)
Interpersonal relationship	475 (7.7)
Study	434 (7.0)
Career	640 (10.3)
Traumatic experience	237 (3.8)
Physical health	132 (2.1)
Personal development	303 (4.9)
Sexual orientation/Gender distress	53 (0.9)
Addictive behavior	53 (0.9)
Social unrest	29 (0.5)
Covid-19	44 (0.7)
Others	325 (5.2)
K6 score, mean (SD)	14.53 (4.61)
Self-injury or suicidal ideation, n (%)	
Yes	2123 (34.2)
No	4084 (65.8)
Age group, n (%)	
Secondary school student	1452 (23.4)
University student	914 (14.7)
Non-student youth	2097 (33.8)
Middle-aged	570 (9.2)
Unknown	1174 (18.9)
Sex, n (%)	
Male	1305 (21.0)
Female	3460 (55.7)
Unknown	1442 (23.3)
Communication channel, n (%)	
Anonymous portal	5212 (84.0)
Nonanonymous portal	995 (16.0)
Repeated help-seeker, n (%)	
Yes	2380 (38.3)
No	3827 (61.7)
Premature departure, n (%)	
Yes	1773 (28.6)
No	4434 (71.4)
Queue time, mean (SD)	13.04 (14.71)
Session duration, mean (SD)	63.31 (30.89)

### GMM for Help-Seeker Sentiment

As shown in Table 4, a 3-class GMM was selected based on multiple criteria including AIC, BIC and maximum log-likelihood. Parameter estimates for the 3-class model are provided in Table S2 in Multimedia Appendix 1.

**Figure 2.**
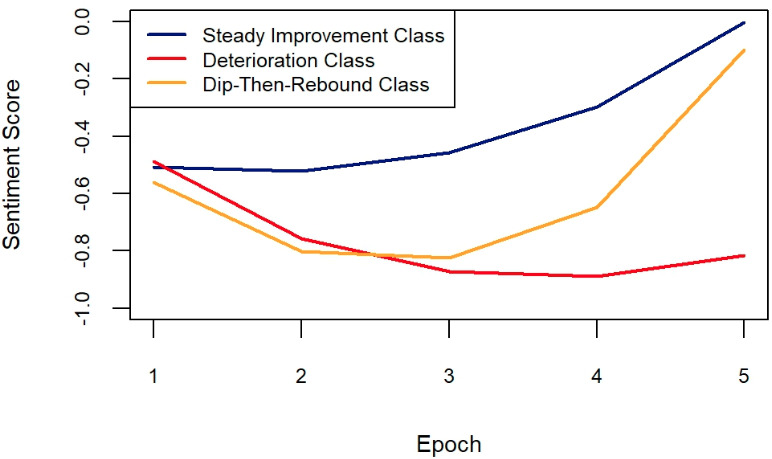
Three-class growth mixture model for help-seeker sentiment.

Figure 2 illustrates the in-session sentiment trajectories for the three latent classes. Among the 6207 sessions, 1171 sessions (18.9%) were clustered as Class 1, which was labeled as the “Steady Improvement Class.” This class displayed stable sentiment from the first epoch to the second epoch, followed by a gradual increase from the second to the fifth epoch.

**Table 4.** Fit indices of different solutions.

Number of classes	AIC^a^	BIC^b^	Maximum log-likelihood
1	17167.18	17214.31	−8576.59
2	15742.33	15816.40	−7860.17
3	15131.36	15232.36	−7550.68
4	15155.75	15283.68	−7558.87
5	15786.11	15940.98	−7870.05
6	15255.12	15436.93	−7600.56

a AIC: Akaike’s Information Criterion.

b BIC: Bayesian Information Criterion.

Class 2, the “Deterioration Class,” consisted of 1119/6207 sessions (18.0%). This class is characterized by a decline in sentiment scores from the first epoch through the third epoch, followed by a relatively flat trajectory from the third to the fifth epoch.

Class 3, the “Dip-Then-Rebound Class,” consisted of 3917/6207 sessions (63.1%). This class reflects a more typical sentiment trajectory of help-seekers in counseling. Help-seekers showed increasingly negative sentiment from the first to the second epoch, a plateau from the second to the third epoch, an uptick from the third to the fourth epoch, and a more pronounced improvement in sentiment from the fourth to the fifth epoch.

### Association of Variables With Trajectory Class

#### Steady Improvement Class Versus Dip-Then-Rebound Class

**Figure 3.**
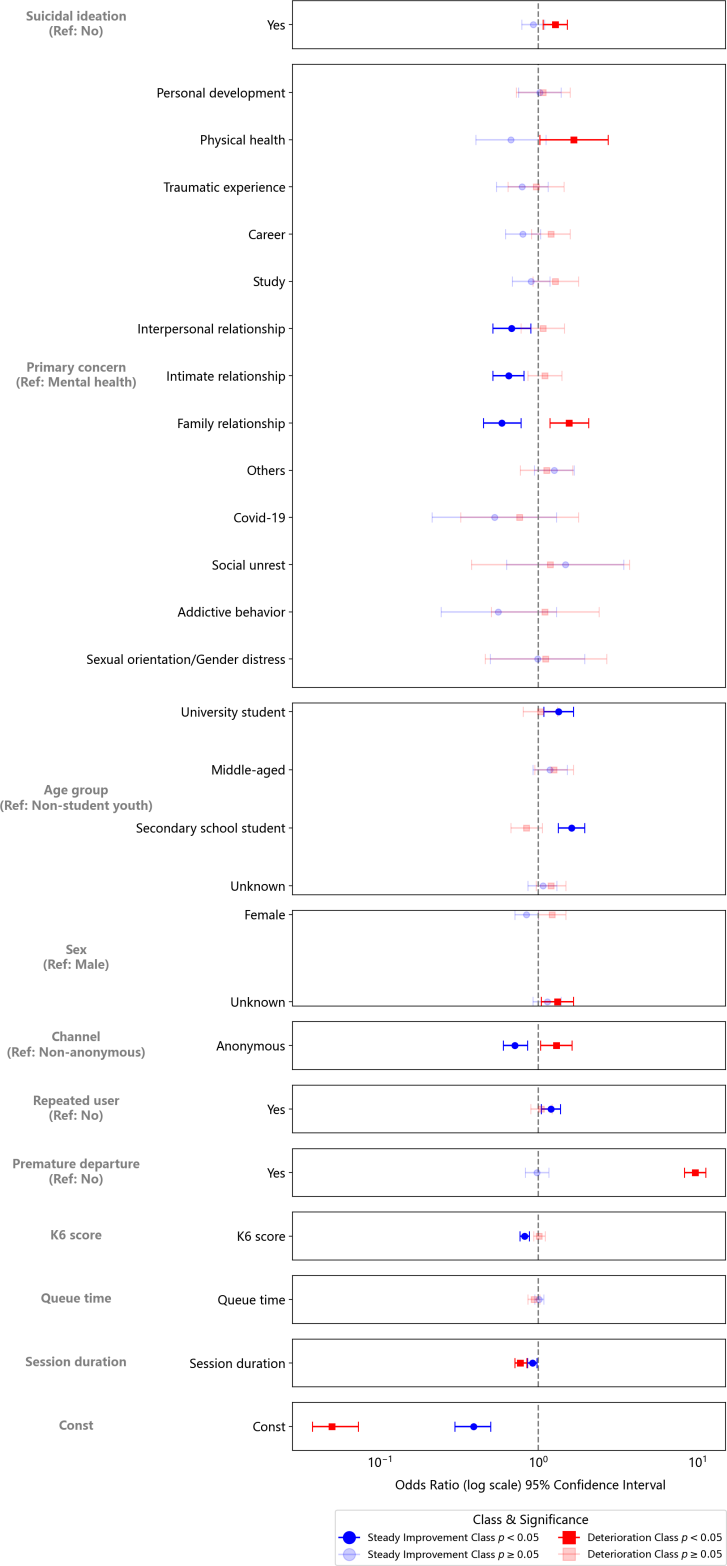
Multinomial logistic regressions of trajectory class membership using variables for a three-class growth mixture model of help-seeker sentiment.

The Dip-Then-Rebound Class, which was the largest of the three, was used as the reference group in the multinomial logistic regression. As exhibited in Figure 3 and the Table S3 in Multimedia Appendix 1, compared with the Dip-Then-Rebound Class, help-seekers in the Steady Improvement Class were less likely to report “Family relationship” (OR 0.59, 95% CIs 0.45-0.78, *P*<.001), “Intimate relationship” (OR 0.65, 95% CIs 0.52-0.81, *P*<.001), or “Interpersonal relationship” (OR 0.68, 95% CIs 0.52-0.90, *P*=.01) as their primary concern. They were more likely to be secondary school (OR 1.62, 95% CIs 1.34-1.95, *P*<.001) or university students (OR 1.34, 95% CIs 1.08-1.67, *P*=.01). Furthermore, they were less likely to access the service via the anonymous portal (OR 0.71, 95% CIs 0.60-0.85, *P*<.001), reported lower K6 scores (OR 0.82, 95% CIs 0.76-0.88, *P*<.001), and had shorter session duration (OR 0.92, 95% CIs 0.85-0.98, *P*=.01). They were also more likely to recontact the service after their first visit (OR 1.20, 95% CIs 1.04-1.38, *P*=.01).

#### Deterioration Class Versus Dip-Then-Rebound Class

Compared with those in the Dip-Then-Rebound Class, members of the Deterioration Class were more likely to report suicidal ideation (OR 1.28, 95% CIs 1.07-1.52, *P*=.006), and cite “Family relationship” (OR 1.56, 95% CIs 1.19-2.08, *P*=.002) or “Physical health” (OR 1.67, 95% CIs 1.02-2.74, *P*=.04) as their primary concern. These help-seekers were also more likely to have unknown gender information (OR 1.32, 95% CIs 1.04-1.67, *P*=.02), and access the service via the anonymous channel (ie, web portal) (OR 1.30, 95% CIs 1.03-1.63, *P*=.03) They were much more likely to depart prematurely (OR 9.76, 95% CIs 8.33-11.36, *P*<.001), and had shorter session duration (OR 0.77, 95% CIs 0.71-0.84], *P*<.001).

## Discussion

### Principal Findings

This study identified 3 distinct in-session sentiment trajectories among help-seekers using a text-based counseling service. The 3 classes—steady improvement (1171/6207, 18.9%), deterioration (1119/6207, 18.0%), dip-then-rebound (3917/6207, 63.1%)—demonstrate significant heterogeneity in how help-seekers’ emotional states evolve during a session. Furthermore, several help-seeker characteristics were associated with membership in each class, sentiment trajectories can serve as an indicator for understanding and tailoring interventions within online counseling contexts. These findings address a gap in the literature regarding practical methods for monitoring help-seekers’ psychological states and the counseling process under conditions of high anonymity [5-8].

### Interpretation of the Three Trajectories

Help-seekers in the steady improvement class exhibited stable sentiment in early stages (ie, the first 3 epochs), followed by a gradual improvement thereafter. Their relatively lower psychological distress scores support the notion that they entered the service with less urgent concerns. For counselors managing multiple sessions simultaneously, early identification of this stable-to-positive trajectory could permit more efficient resource allocation. Shorter sessions or timely referrals may suffice if the help-seeker’s needs do not require intensive intervention.

In contrast, help-seekers in the deterioration class exhibited continuously worsening of sentiment throughout the session. This pattern was associated with more frequent suicidal ideation, higher likelihood of family or physical health-related issues, and a high rate of premature departure. As suggested by Xu et al [26], premature departure could indicate dissatisfaction with the counseling session. This class might represent a potential mismatch between help-seeker needs and the interventions provided. In addition, these help-seekers were more likely to access the service via the anonymous communication channel (ie, web portal), and less likely to disclose their gender, which can undermine the counselor’s ability to tailor support. Early detection of such negative trends could enable counselors to potentially shift their strategies or provide specialized resources before sentiments deteriorate further.

The largest group, the dip-then-rebound class, displayed a more typical counseling progression where the help-seeker initially expressed increasing negativity during initial problem exploration but rebounded in later phases, likely reflecting effective therapeutic engagement [12]. This pattern indicates that, for the majority of help-seekers, text-based counseling can help improve their affect—and potentially reflect successes in addressing their presenting issues—over the course of a single session.

### Practical Applications and Future Directions

The findings have significant practical applications. Specifically, our approach laid the groundwork for predictive tools that could monitor in-session sentiment and forecast which pattern a help-seeker might follow. Such tools could enable counselors to respond proactively in real time.

For example, Table 5 highlights 3 exemplar sessions—one from each trajectory—showing how counselors could adapt their strategies based on emerging sentiment patterns. The help-seekers in the 3 sessions expressed a similar level of negativity in the first epoch of each session. In the session representing the steady improvement class, detecting a reduced negativity by the second epoch may prompt the counselor to conduct and close the session more quickly, especially if they judge the help-seeker’s concerns and distress to be relatively mild. In the session depicting the deterioration class, heightened negativity observed by the fourth epoch could trigger escalating the case to a more experienced counselor or suggesting immediate external support. In the session showcasing the dip-then-rebound class, noticing the rebound in sentiment around the fourth epoch indicates alignment with typical counseling protocols, as the help-seeker appears to benefit from interventions.

Future predictive model might track real-time, message-by-message sentiment rather than segmenting sessions into discrete epochs, thus providing just-in-time assistance for counselors. By identifying likely trajectory earlier, counselors can be more proactive and adaptive in their intervention strategies, ensuring appropriate and timely support.

**Table 5.** Sample open-up sessions illustrating potential application of sentiment trajectories.

Epoch	Steady improvement class		Deterioration class		Dip-then-rebound class	
	Summary	Sentiment score	Summary	Sentiment score	Summary	Sentiment score
1	The help-seeker shared guilt and confusion over a recent breakup.	−0.778	The help-seeker expressed initial frustration and fatigue due to a combination of work stress and post-Covid symptoms.	−0.667	The help-seeker expressed sadness due to various stressors.	−0.750
2	Negativity decreased as the help-seeker felt relieved after venting. The counselor showed empathy and reassurance.	−0.556	Negativity increased as the help-seeker described a long commute and the increasing work pressure.	−0.714	Negativity peaked as the help-seeker showed feelings of hopelessness and doubts about their ability.	−1.000
3	Negativity continued to decrease as the help-seeker began to minimize the severity of their concern. The counselor continued to validate emotions and reassure the help-seeker.	−0.556	The help-seeker showed increasing helplessness about balancing health, workload, and financial stress, despite the counselor’s proposed solutions, such as contacting school counselors.	−0.833	Negativity remained low, and the counselor started to guide the help-seeker to consider alternative perspectives.	−1.000
4	Negativity significantly reduced as the help-seeker exhibited confidence in managing her issue independently. The counselor affirmed the help-seeker’s ability and constantly showed emotional support.	−0.111	The help-seeker explicitly expressed feelings of sadness and hopelessness, blaming themselves for their current situation. The counselor suggested seeking professional therapy but struggled to reduce the help-seeker’s distress.	−0.857	Negativity began to decrease as the help-seeker agreed on the proposed suggestions and expressed gratitude to the counselors.	−0.400
5	The help-seeker demonstrated readiness to solve the problem and positivity.	−0.111	The help-seeker’s distress remained unresolved despite the counselor’s efforts.	−0.857	Sentiment turned positive as the help-seeker felt optimistic about their future.	0.600

Although this study provided valuable insights into help-seekers’ emotional processes in text-based counseling, it also has several limitations that warrant attention in future research. First, while ChatGPT was highly accurate and computationally effective for labeling sentiments, its use of only 3 broad sentiment categories (positive, neutral, and negative) lacks granularity. Future studies could explore more advanced sentiment analysis models that capture arousal or emotional intensity, potentially leading to deeper insights and more precise interventions.

Second, the dataset was limited to help-seekers aged 11‐35 years old from a specific online text-based counseling service in Hong Kong, which might limit the generalizability of the findings to other populations and contexts. Further implementation and validation of this model in diverse settings are needed to enhance its broader applicability.

Third, only first-time visit sessions were included for analysis, which warrants future investigation on sentiment changes within repeated visit sessions. As Open Up was designed to primarily provide single-session intervention, the decision to only include first-time visits ensures that the analysis aligns with and is representative of the service’s scope and excludes potential confounding factors arising from recontacts. Although this criterion focused our analysis on first-session experiences, it also highlights the need for future research that could incorporate subsequent repeated visits. This could allow us to assess sentiment changes across repeated sessions [15], examine between-sessions dynamics and longer-term outcomes over multiple counseling engagements, and better understand repeated help-seekers’ profiles.

Fourth, exclusion of sessions without presession survey responses may introduce selection bias. As exhibited in Table S1 in Multimedia Appendix 1, excluded sessions differed from included ones in several demographic and session-related characteristics, although effect sizes for most variables were small to moderate. Importantly, as psychological profile data were not available for excluded sessions, potential differences in key variables such as distress or suicidal ideation could not be compared between the 2 groups. Finally, this study did not analyze the counselor’s sentiment trajectory. As counseling is an interactive and reciprocal process, examining both counselor and help-seeker sentiments in tandem could yield richer insights into the counseling relationship and outcome efficacy.

Finally, this study did not analyze the counselor’s sentiment trajectory. As counseling is an interactive and reciprocal process, examining both counselor and help-seeker sentiments in tandem could yield richer insights into the counseling relationship and outcome efficacy.

### Conclusion

This study used a GMM and identified three latent groups of help-seekers with distinct in-session sentiment trajectories in an online text-based counseling platform in Hong Kong: steady improvement, deterioration, and dip-then-rebound. Multinomial logistic regression revealed help-seeker characteristics associated with each trajectory. Specifically, the deterioration class was characterized by more suicidal ideation, higher proportions of family or physical health-related issues, a higher tendency to withhold demographic information and prematurely exit the session. These findings could inform the future development of predictive models that dynamically track help-seekers’ emotional states in real-time, enabling counselors to adapt interventions swiftly and appropriately and, ultimately, enhance the effectiveness of online text-based counseling.

## Supplementary material

10.2196/75091 Multimedia Appendix 1 Supplementary materials.

## Abbreviations

AI: artificial intelligence
GMM: Growth Mixture Model
LIWC: Linguistic Inquiry and Word Count
NLP: natural language processing
