# Supplementary material for: Assessing Heterogeneity in Sentiment Changes in Text-Based Counseling: Latent Class Trajectory Analysis
Source: J Med Internet Res. 2025 Sep 5;27:e75091. doi: 10.2196/75091 (PMC12413188; doi:10.2196/75091)
Supplement: Multimedia Appendix 1 [file jmir-v27-e75091-s001.docx]

# Supplementary Materials

Contents

[Comparison of sessions without pre-session survey response and sessions included for analysis 2](#_Toc203987869)

[Parameter estimates of GMM with three classes 4](#_Toc203987870)

[Multinomial logistic regression output of three-class growth mixture model 4](#_Toc203987871)

## Comparison of sessions without pre-session survey response and sessions included for analysis

To assess potential selection bias resulting from the exclusion of sessions lacking pre-session survey responses, we compared the distribution of available session-related and demographic variables between sessions that were excluded due to lack of pre-session survey response (n = 8,906) and those included in the primary analysis (n = 6,207). Variables examined include communication channel, premature departure, repeated help-seeker, queue time, session duration, age group, and gender.

The results highlighted several statistically significant differences between excluded and included sessions. Notably, excluded sessions had higher rates of premature departure (46.45% vs. 29.58%, $\chi^{2}$ = 434.93, p < .001, OR = 0.48) and repeated help-seeking (52.30% vs. 38.34%, $\chi^{2}$ = 285.84, p < .001, OR = 0.57), longer queue times (M = 17.50, SD = 33.34 vs. M = 13.04, SD = 14.71; F = 11.16, p < .001, Cohen d = 0.16), and shorter session durations (M = 53.07, SD = 47.89 vs. M = 63.31, SD = 30.89; F = 15.97, p < .001, Cohen d = 0.25). In addition, excluded sessions had a higher proportion of unknown responses in age group (37.20% vs. 17.65%, $\chi^{2}$ = 782.35, p < .001, Cramér’s V = 0.23) and gender (44.46% vs. 22.55%, $\chi^{2}$ = 742.52, p < .001, Cramér’s V = 0.22). Although these differences were statistically significant, the effect sizes for most variables were small to moderate, suggesting that while selection bias is present, its practical impact might be limited for these variables. However, variables related to user’s psychological profile measured in pre-session survey (e.g., distress, suicidal ideation) remain unavailable in excluded sessions; therefore, we could not compare those variables between the two groups.

Supplementary Table 1. Comparison of statistics between sessions without pre-session survey response and sessions included for analysis.

| Variables | | Excluded sessions  ($n=8,906$) | Included sessions  ($n=6,207$) | $F$/$\chi^{2}$ | $p$-value | Effect size |
| --- | --- | --- | --- | --- | --- | --- |
| Anonymous channel  $n$(%) | | 7,361 (82.65) | 5,212 (83.97) | 4.45 | .003 | 1.10^a^ |
| Premature departure  $n$(%) | | 4,137 (46.45) | 1,836 (29.58) | 434.93 | < .001 | 0.48^a^ |
| Repeated help-seeker  $n$(%) | | 4,658  (52.30) | 2,380  (38.34) | 285.84 | < .001 | 0.57^a^ |
| Queue time  Mean (SD) | | 17.50  (33.34) | 13.04  (14.71) | 11.16 | < .001 | 0.16^b^ |
| Session duration  Mean (SD) | | 53.07  (47.89) | 63.31  (30.89) | 15.97 | < .001 | 0.25^b^ |
| Age group  $n$(%) | Secondary school student | 1,095  (12.30) | 1,376  (23.69) | 782.35 | < .001 | 0.23^c^ |
|  | University student | 1,020  (11.45) | 876  (15.08) |  |  |  |
|  | Non-student youth | 2,782  (31.24) | 1,999  (34.41) |  |  |  |
|  | Middle-aged | 696  (7.81) | 533  (9.18) |  |  |  |
|  | Unknown | 3,313  (37.20) | 1,025  (17.65) |  |  |  |
| Gender  $n$(%) | Male | 1,484  (16.66) | 1,233  (21.23) | 742.52 | < .001 | 0.22^c^ |
|  | Female | 3,462  (38.87) | 3,266  (56.22) |  |  |  |
|  | Unknown | 3,960  (44.46) | 1,310  (22.55) |  |  |  |

Note. ^a^ Odds Ratio; ^b^ Cohen’s d; ^c^ Cramér’s V

## Parameter estimates of GMM with three classes

Supplementary Table 2. Parameter estimates of GMM with three classes.

| Number of classes | BIC | n (%) | Intercept | Slope | Quadratic slope |
| --- | --- | --- | --- | --- | --- |
| 3 | 15232.36 | 1,171 (18.9) | 0 | -0.40 | 0.12 |
|  |  | 1,119 (18.0) | 1.15 | -1.58 | 0.21 |
|  |  | 3,917 (63.1) | 1.26 | -2.11 | 0.40 |

## Multinomial logistic regression output of three-class growth mixture model

Supplementary Table 3. Multinomial logistic regression output of three-class growth mixture model.

|  | | **Steady Improvement Class (vs. Dip-Then-Rebound Class)** | | | **Deterioration Class (vs. Dip-Then-Rebound Class)** | | |
| --- | --- | --- | --- | --- | --- | --- | --- |
| **Characteristics** | | **OR** | **95% CIs** | **p-value** | **OR** | **95% CIs** | **p-value** |
| Primary concern  (vs. Mental health) | Family relationship | **0.59** | **(0.45, 0.78)** | **< .001** | **1.56** | **(1.19, 2.08)** | **.002** |
|  | Intimate relationship | **0.65** | **(0.52, 0.81)** | **< .001** | 1.10 | (0.86, 1.40) | .454 |
|  | Interpersonal relationship | **0.68** | **(0.52, 0.90)** | **.007** | 1.07 | (0.78, 1.46) | .678 |
|  | Study | 0.90 | (0.68, 1.19) | .459 | 1.28 | (0.92, 1.79) | .140 |
|  | Career | 0.80 | (0.62, 1.03) | .085 | 1.20 | (0.90, 1.58) | .204 |
|  | Traumatic experience | 0.79 | (0.54, 1.15) | .217 | 0.97 | (0.64, 1.45) | .868 |
|  | Physical health | 0.67 | (0.40, 1.12) | .125 | **1.67** | **(1.02, 2.74)** | **.041** |
|  | Personal development | 1.02 | (0.75, 1.39) | .892 | 1.07 | (0.73, 1.58) | .719 |
|  | Sextual orientation/Gender distress | 0.99 | (0.50, 1.95) | .975 | 1.11 | (0.46, 2.69) | .810 |
|  | Addictive behaviour | 0.56 | (0.24, 1.30) | .175 | 1.10 | (0.51, 2.41) | .809 |
|  | Social unrest | 1.48 | (0.63, 3.46) | .368 | 1.19 | (0.38, 3.75) | .766 |
|  | Covid-19 | 0.53 | (0.21, 1.30) | .164 | 0.76 | (0.32, 1.78) | .528 |
|  | Others | 1.26 | (0.94, 1.68) | .118 | 1.13 | (0.77, 1.65) | .535 |
| K6 score | | **0.82** | **(0.76, 0.88)** | **< .001** | 1.01 | (0.93, 1.11) | .788 |
| Self-injury or suicidal ideation  (vs. No) | Yes | 0.93 | (0.79, 1.09) | .368 | **1.28** | **(1.07, 1.52)** | **.006** |
| Age group  (vs. Non-student youth) | Secondary school student | **1.62** | **(1.34, 1.95)** | **< .001** | 0.84 | (0.67, 1.06) | .139 |
|  | University student | **1.34** | **(1.08, 1.67)** | **.007** | 1.03 | (0.80, 1.32) | .811 |
|  | Middle-aged | 1.18 | (0.92, 1.52) | .189 | 1.25 | (0.95, 1.67) | .113 |
|  | Unknown | 1.07 | (0.86, 1.31) | .551 | 1.20 | (0.97, 1.49) | .093 |
| Gender  (vs. Male) | Female | 0.84 | (0.71, 1.00) | .051 | 1.22 | (1.00, 1.49) | .052 |
|  | Unknown | 1.14 | (0.92, 1.39) | .222 | **1.32** | **(1.04, 1.67)** | **.021** |
| Communication channel  (vs. Non-anonymous channel) | Anonymous channel | **0.71** | **(0.60, 0.85)** | **< .001** | **1.30** | **(1.03, 1.63)** | **.025** |
| Repeated help-seeker  (vs. No) | Yes | **1.20** | **(1.04, 1.38)** | **.011** | 1.04 | (0.90, 1.22) | .587 |
| Premature departure  (vs. No) | Yes | 0.98 | (0.83, 1.16) | .820 | **9.76** | **(8.33, 11.36)** | **< .001** |
| Queue time | | 1.01 | (0.95, 1.08) | .714 | 0.94 | (0.86, 1.02) | .144 |
| Session duration | | **0.92** | **(0.85, 0.98)** | **.014** | **0.77** | **(0.71, 0.84)** | **< .001** |

Note. OR = Odds ratio; 95% CIs = 95% confidence intervals
